# The evolutionary origins of dementia

**DOI:** 10.1177/25424823261470053

**Published:** 2026-07-21

**Authors:** Jonathan Stone, Daniel M Johnstone, Rebecca S Mason, John Mitrofanis, Stephen R Robinson

**Affiliations:** 1 Faculty of Medicine and Health, 638093 University of Sydney, Sydney, Australia; 2 School of Biomedical Sciences and Pharmacy, 5982 University of Newcastle, Newcastle, Australia; 3 School of Life and Environmental Sciences, Charles Perkins Centre, 638093 University of Sydney, Sydney, Australia; 4 27015 Université Grenoble Alpes, Fonds de Dotation, Grenoble, France; 5 Institute of Ophthalmology, University College London, London, UK; 6 School of Health and Biomedical Sciences, 5376 RMIT University, Bundoora, Australia; 7 Institute for Breathing and Sleep, 3805 Austin Health, Heidelberg, Australia

**Keywords:** Alzheimer's disease, blood-brain barrier, dementia, elastin, evolution, glutamate, heart, heme, hemoglobin, neurogenesis, oxygen, vascular aging

## Abstract

Evidence has grown that the core pathology of age-related dementias, including Alzheimer's disease, is microvascular—small, symptomless bleeds from cerebral capillaries, accelerated by the hypertension of age. Each such bleed, this evidence suggests, damages a patch of brain, causing the death of neurons. This review asks how this vulnerability of the brain emerged from evolution, to cause age-related dementia? And has the vulnerability spurred the evolution of mitigating mechanisms? It is argued that seven features of human evolution contribute to the cause of dementia: the dependence of our tissues, especially the brain, on oxygen; the toxicity of the molecule (heme) that binds to oxygen to enable its transport; the pulsatility of the heart; the danger of glutamate as an excitatory neurotransmitter; the irreparability of elastin; the low level of adult neurogenesis in humans; and the breakdown, in late life, of the blood-brain barrier. Also discussed are mechanisms that have evolved to counter these threats to the brain, including the generation of a reserve of brain tissue. We argue that the vulnerabilities of the brain to damage that accumulates throughout life, and leads to dementia in the aged, were established early in the evolution of vertebrates. Several can be understood as unavoidable yet damaging outcomes of highly advantageous steps in evolution. Human cognition fails when and because, with age, the damage that results from these vulnerabilities overwhelms the evolved protections and exhausts any reserve of brain tissue. Implications of these vulnerabilities for the delay and management of dementia are discussed.

## Introduction

It is not common to analyze diseases as products of evolution but, even so, this essay examines human dementia in those terms, because ‘dementia’ is more a prognosis than a diagnosis. The designation tells us little about what has happened to cause the cognitive loss and brain shrinkage that are among the diagnostic criteria, because the literature on cause, although extensive, is unsettled. The designation does tell us, with some certainty, that the sufferer is facing progressive cognitive loss and brain shrinkage, despite the treatments available, and that the condition will be with them to their death. Lifestyle and genetic risk factors for Alzheimer's disease have been identified in detail, without the pathogenesis of the condition being widely agreed.

Many ideas of the cause of dementias like Alzheimer's disease have been proposed, the best known, though challenged,^1–3^ being the amyloid cascade hypothesis, which posits the cause as the appearance in the aging brain of proteins that are toxic to it, particularly the peptide amyloid-β (Aβ, reviewed in^4^). Even in those terms, however, the appearance of the proteins/peptides considered causative is itself unexplained, leaving much to be debated; and the debate has gained nuance from evidence that Aβ has significant trophic and protective roles in the brain. Aβ may be a protective first-responder molecule expressed by the brain when and where it is damaged.^5,6^ Treatments targeting Aβ, on the assumption that its toxicity is causal, have received approval in the USA (https://www.fda.gov/news-events/press-announcements/fda-converts-novel-alzheimers-disease-treatment-traditional-approval; https://www.dementia.org.au/media-centre/media-releases/drug-approval-promising-people-early-alzheimers) and, very recently, in Australia, but not yet in other jurisdictions. Such treatments do lower the ‘burden’ of Aβ in the brain, but the clinical value of this reduction is presently disputed.^1,2^

In recent reviews, with colleagues and in parallel with others (e.g.,^7^), we have argued that several age-related forms of dementia (pugilistica, chronic traumatic encephalopathy, combat-related, Alzheimer's disease) share a cause in the hemorrhage of cerebral capillaries, caused as a function of age by the stiffening of the walls of the aorta^8–13^ and the great distributing arteries, and accelerated by head trauma.^14^ That stiffening and its effects of increasing systolic and pulse pressures are well established, uncontroversial. The evidence supporting the idea that several forms of dementia are driven by the age-intensified pulse is epidemiological (that hypertension increases the risk/brings forward the onset of dementia^15–20^), neuropathological (that capillary bleeding is evident in the aging brain and causes the formation of Aβ^+^ plaques^21–23^); toxicological (that blood, particularly free hemoglobin, is toxic to neurons^24^); and interpretational (that Aβ is not a self-toxin, but a first-responder molecule generated by neurons to protect themselves from the toxicity of heme and from opportunistic pathogens^5,6^). Capillary hemorrhage, in this understanding, is caused by the internal trauma of the aging pulse and accelerated by external trauma, especially to the head.^14^

The growing prevalence of dementia in developed societies could be regarded as a triumph of medical science, the result of the protection of the mid-aged from non-infectious conditions, like cardiovascular diseases and cancers. But dementia is an awful condition, which must be addressed. And an important step is to understand it, including its origins in evolution. We have, in previous essays, traced the causes of these age-related dementias from cognitive loss back to the loss of elastin in the great distributing arteries,^9,10^ and temporally back from old age, when they are diagnosed, to mid-life^25^ when hardening of the aorta and subtle cognitive loss can be detected in everyone. We here seek the cause of dementia along another dimension of time, the evolution of our species. All aspects of the body are shaped by evolution; what then are the features of the evolution of mammals that have made the human brain vulnerable to late-life degeneration, as evident in what some have called a ‘plague’ among the old?^26^

## Evolutionary causes of dementia

The distinction between immediate (individual lifetime) and evolutionary causes of a disease or condition is perhaps novel; it seems worth exploring because the identification of evolutionary causes can increase our understanding and shape our expectations. We argue below for the recognition of 7 ‘evolutionary causes’ of age-related dementias in humans.

### Three evolutionary causes related to oxygen

Vertebrates have evolved to be obligate aerobes, dependent on a constant availability of oxygen. Recruiting oxygen into their metabolism allowed vertebrates to utilize much more of the energy available in carbohydrates, when they are oxidized; that oxidation was made possible by the evolution of electron transport chains,^27^ in particular the oxidative phosphorylation pathways found in vertebrate mitochondria. These energy-producing pathways are considered to have evolved when early, anaerobic forms of life ‘polluted’ the earth's atmosphere with the oxygen they produced from carbon dioxide, creating a marked and sustained rise in atmospheric oxygen some 2.4 billion years ago (the ‘great oxygen event’ discussed by^28^). The energy made available by oxidative phosphorylation made possible the evolution of more complex and energetic animals.

Among the tissues of the vertebrate body, oxygen-dependence is greatest for the brain, because of the brain's high metabolic activity per unit weight (∼10-fold higher than the average of all other tissues). That high metabolic rate arises from the evolution of cells (neurons) that signal to each other, the basis of any nervous system. In neurons, the ionic pumps present in the membranes of probably all vertebrate cells are scaled up significantly, to set and maintain the resting membrane potential of nerve cells (∼-70mv), which is then modulated (using for example voltage-gated channels) by signals received from other neurons. These modulations of membrane potential are the basis of all neural signaling; the signaling requires maintenance of the neurons’ resting potential; this maintenance is achieved by ionic pumps; the pumps require ATP and the production of ATP requires oxygen, in the oxidative phosphorylation pathways. A complex nervous system creates a great survival advantage for vertebrates, but the brain's demand for oxygen brings a survival disadvantage, making anoxia and hypoxia threats throughout individual life. Some authors have argued^29^ that the intense development of association cortex in primates has intensified the oxygen-dependence of the cerebral cortex in humans.

This disadvantage is, the success of the vertebrates makes clear, outweighed in evolutionary terms by the value of a nervous system, but the disadvantage does not disappear. It becomes evident when, for example, we face any form of asphyxiation and when we age and our cerebral blood vessels fail, causing strokes (when large vessels fail) or, as in dementia, when the vessels that fail are too small to cause symptoms.^6,10,30^

***The toxicity of heme.*** The molecule that, in vertebrates, evolved to bind oxygen in the lungs and transport it to tissue is heme, described as ‘an iron-protoporphyrin complex’ which serves as a ‘key prosthetic group of oxygen-binding proteins’ such as hemoglobin, myoglobin and neuroglobin.^31^ It seems remarkable that a molecule so essential to vertebrate tissues is, when free in those tissues, seriously toxic to them. Its toxicity was first recognized in the hemolytic anemias (thalassemia, sickle cell disease) and in the lethal ‘cerebral’ stage of malaria,^32–34^ in all of which the breakdown of circulating red cells releases hemoglobin into the blood, causing widespread morbidity. The symptoms of sickle cell disease include chronic pain, pain crises, an increased risk of stroke and damage to the lung, retina and kidneys. In people without these conditions, recent authors have argued that heme is damaging to the brain in hemorrhagic stroke^24,35^ and in the dementias.^6^ Its toxicity is also evident in how it is chaperoned around the body, bound to a globin protein, internalized in red cells and the red cells kept out of the brain by the blood-brain barrier; until there is a hemorrhage. Scientists who have traced the evolution of the erythrocyte have emphasized its achievements: by containing hemoglobin in a specialized cell, they stress, the amount of hemoglobin in the blood can reach high levels (typically 13–16 g/100 ml in humans, https://www.redcrossblood.org/donate-blood/dlp/hematocrit.html), increasing oxygen delivery without making the plasma viscous and the breakdown of hemoglobin by oxidative mechanisms and by the kidney is minimized, increasing its half-life. Uniquely among mammalian cells, erythroblasts have evolved to separate into two cells as they differentiate, one daughter cell (the erythrocyte, containing hemoglobin) will serve as the oxygen transporting cell for typically 120 days; the other, containing the nucleus, is called a pyrenocyte and is quickly phagocytosed.^36^ The small size of the erythrocyte facilitates, these authors point out, the ready passage of oxygen, bound to hemoglobin internalized into red cells, through capillaries, optimizing the delivery of oxygen to tissues.

But, when that highly evolved mechanism breaks down, when a vessel bleeds and the erythrocytes lyse, the free hemoglobin breaks down to toxic forms that kill neurons. The evolutionary question then is—why did there not evolve an oxygen-carrier less toxic than heme? Authors who have addressed this question point to three issues: First, oxygen became important in the respiration of animals (and plants) during the ‘great oxygenation event’ of the atmosphere referred to above. In those conditions, an oxygen-carrier molecule gave a huge survival advantage, enabling oxygen to be transported to the sub-millimeter level in the tissues of large animals. Second, that carrier needed (obviously) to bind oxygen, which ionic iron can don (oxygen and iron bind by ‘polar forces’ intrinsic to their chemistry, https://en.wikipedia.org/wiki/Hemoglobin), and iron was abundant. Third, these investigators point out that the catalytic activity of iron is needed to bind oxygen. The reactivity of iron that made it valuable in the transport of oxygen has a damaging impact on the proteins that make up body tissues^37,38^; the toxicity of heme cannot be separated from its evolutionary value.

On a comparative note, hemoglobin is ‘the single, dominant, essentially universal respiratory pigment of the vertebrates’.^39^ In some sea- and land-dwelling invertebrates, oxygen is transported by a different molecule, hemocyanin, in which copper is the oxygen-binding metal. Hemocyanin is less toxic than hemoglobin, allowing it to be free in the circulating hemolymph. Like hemoglobin, hemocyanin evolved to have several oxygen-related roles, beyond oxygen transport.^40^ On the other hand, it is less efficient: its capacity to carry and deliver oxygen has been estimated at 1/10th that of hemoglobin (https://scipedia.bohrium.com/en/sciencepedia/feynman/keyword/hemolymph_vs_blood).

***The pulsatility of the heart.*** After earlier observers had understood parts of the role of the heart as the pumper of blood, it was William Harvey, in his 1628 *De Motu Cordis*,^41^ who demonstrated that the mammalian heart is a double-pump, driving both the pulmonary and systemic circulations,^42^ in parallel and interconnected. The clarity of his demonstration gave impetus to the disciplines of body structure and function (so anatomy and physiology), but the mundane truth, that the heart is a pump, was at odds with a more poetic understanding that the heartbeat is a spirit of life, its weakness a harbinger of death; that the heart thumps into our consciousness when we are exercising or aroused, giving the heart an entirely unscientific place in idioms of courage, adventure and romance. Harvey was aware of a tension between the romance and the science of the heart and wrote, in his Chapter 5:

*Whether or not the heart, besides propelling, distributing and giving motion locally to the blood, adds anything else to it – heat, spirit, perfection – must be investigated later, and decided on other grounds.*

In the grim idiom of this essay, of old age and dementia, the heart emerges in another role: it becomes with age the organ that pummels our cerebral cortex into a thinning, hemorrhage-ridden patchwork of Aβ+ plaques (the modern term for what Alzheimer^43^ called *Herde* or ‘foci’) and surviving circuitry (see for example, Figures 1–6 in^22^). Over the life of any individual, the heart is, at every age, an essential pump and a sign of life; in the adventures of youth, it is the thump of excitement; in sober adulthood it is the steady beat of survival; and then, in those who survive long, it becomes the destroyer of the brain. For the old, and for those who wish to survive to be old (so maybe all of us), the reality is bleak. If the heart stops, we die quickly; if it keeps beating, we die more slowly, of dementia and its complications.

Taking an overview, a biologist might then ask: would blood flow effectively through body tissues under steady pressure, driven by a continuous pressure pump? So that the heart could avoid this last, destructive role? A search of the literature on the evolution of the heart indicates that a heart-pump of some sort became necessary as animals evolved to be larger than an earthworm, and that there seem to be no instances of a non-pulsatile heart in the world of animals, certainly not in vertebrates. A pulsatile heart is thus the evolved solution to the need for a pump to drive blood through the tissues of larger organisms. Some invertebrates have evolved multiple hearts; the octopus, for example, has three, a single ‘main’ heart and two auxiliary hearts pumping blood through its two gills. But all three are pulsatile.

Relevant in this context is the experience of cardiac surgeons and medical engineers in the development of ‘artificial hearts’, designed initially to keep patients with advanced heart failure alive until a human heart became available for transplantation. Left ventricular assist devices (LVADs) are considered to have revolutionized the treatment of patients with advanced left ventricular failure.^44^ The first devised were pulsatile,^45^ mimicking the evolved solution, but their large size and low durability led to the development of CF- (continuous flow) LVADs, that overcome these drawbacks.^46^ Tsiouris and colleagues^46^ reported that, among 200 CFLVAD recipients, 94% were still alive at 30d after implantation and 45% at 4 years. They regarded these outcomes as ‘excellent’ for the recipients, though not as good as a transplanted heart. Five years later, Zimpfer and colleagues^47^ reported a survival rate of 51% at 7 years and less formal reports have put the record survival of a patient on an LVAD at over 15 years. Recent improvements to CFLVADs, designed to extend their lifetime to match the patient's,^45^ include ‘mag-lev’ technology (https://www.facs.org/for-medical-professionals/news-publications/news-and-articles/acs-brief/august-6-2024-issue/fellows-implant-world-first-magnetic-levitation-artificial-heart/).

Complications of LVADs are more frequent with continuous flow than with earlier pulsatile devices^48^; but the continuous flow devices are considered more manageable in ways that make them preferable.^49,50^ Their complications are therefore increasingly studied^45^ and have stimulated the further development of CFLVADs,^51^ including the adding of pulsatility to continuous flow,^49^ increasingly with the aim that CFLVADs will be ‘destination therapy’ for sufferers of left ventricular failure.^44^

The sticking point for the evolution of a continuous flow heart seems evident in the mechanism that engineers use—an impeller, spinning on an axle. Wheels and axles seem never to have evolved in biology. The striking point of the reports just considered is that humans can live for years without a beating heart, with blood circulating under continuous pressure, despite the complications that arise. Whether we might ever choose to protect the brain from the pulse, in the hope of delaying dementia, by decommissioning a healthy left ventricle and replacing it with a CFLVAD, remains untested, a leap too far. But we can imagine it.

### One cause related to a neurotransmitter

***Death by excitement: The danger of glutamate.*** It was something of a surprise for neuroscientists when it emerged^52^ that the amino acid glutamate, long known as the taste-enhancer MSG, is the major excitatory neurotransmitter of the human brain. Glutamate's evolution as a neurotransmitter has obviously been successful, but glutamate threatens the brain throughout life because excess levels in the neuropil can cause the death of neurons, by overexcitation. Glutamate levels are much higher (40–60 µM) in blood than in cerebrospinal or brain intracellular fluid (1–10 µM).^53^ Glutamate levels in the brain must be kept low to avoid excitotoxity, and to ensure that glutamate-mediated activity between neurons can operate with a strong signal-to-noise ratio. To this end, the endothelial cells of cerebral blood vessels, and the astrocytes that invest these vessels, actively transport glutamate, against the concentration gradient, out of the brain and into the blood. When this control of glutamate levels in brain tissue is lost, as in an intracerebral hemorrhage, glutamate levels in the affected patch of brain tissue can rise to a level at which the neurons can be killed by over-activation (excitotoxicity^52^). Much study has therefore been directed to glutamate efflux at the blood-brain barrier, including its mechanisms and as a potential site for therapy, for example in stroke.^54–56^ Excitotoxicity has been discussed as a cause of neuron loss in the aging brain and as a cause, therefore, of dementia.^57^ In short, the high levels of glutamate in blood are a constant threat to cerebral neurons.

Could things have evolved otherwise? Presumably so; there might have evolved an excitatory neurotransmitter that was less prevalent in blood. But the reality is that glutamate-induced excitotoxicity is one of the several toxicities that make intracerebral hemorrhage a ‘catastrophe’.^6,35^

### Three causes from failures of repair or protection

***The irreparability of elastin.*** Collagen and elastin are the two major proteins expressed by fibroblasts in the laying down of the body's connective tissues. Collagen comprises ∼50% of skin, while elastin, by contrast, comprises only 0.6–7.9% of most connective tissues.^58^ A second contrast between them is that most of the genesis of elastin occurs prenatally and in early postnatal life, with little or none generated in adulthood; whereas collagen synthesis is a major component of skin repair throughout adult life. And a third contrast is in their half-lives once generated. The half-life of elastin has been estimated at 70 years,^59^ much longer than the half-life of collagen (15 years in skin,^60^ considerably longer in cartilage). The lack of elastin synthesis in adulthood is not understood in evolutionary terms but, as a result of the lack, the elastic modulus of the aortic wall increases several fold` with age, our arteries ‘harden’ and the resultant hypertension is seriously detrimental to quality of life for the aged.^8–12,15–20^

The importance of elastin in the aorta, and in the large distributing arteries, deserves some detail. Our reading of the literature is that, in humans, the tissue richest in elastin is the tunica media of the aorta's wall, where so much is laid down that elastin constitutes 28–32% by dry weight of the wall, a far higher percentage than in skin (0.6% −7.9%; above and see Table 1 in,^61^ which summarizes data from a range of sources), in lung (3–7%) where it is considered important for the lifelong stretch and return of respiration, or in the Achilles tendon of the foot (4.4%). This high proportion of elastin in the aortic wall, and its organization into sublaminae (Figure 1 in^62^), would seem to confirm the importance for species survival of aortic distensibility. By the age of 70 (the half-life of elastin, above) the loss of elasticity in the aorta increases systolic and pulse pressures, contributing to large-vessel strokes^63^ and small-vessel, clinically silent microbleeds,^64–66^ and the pathology that accumulates towards dementia has begun. As one example of this link between the state of the arterial wall and dementia, Liu and colleagues^67^ reported, from postmortem human material, that the extent of elastin loss and of increased rigidity in one of the major brain arteries (the anterior cerebral) was correlated with the cognitive state of the individuals: the arterial wall had deteriorated more in those with a history of dementia.

Investigators have begun work to promote elastin synthesis, including in two models: for the lung failing in chronic obstructive disease, the inhalation of pro-elastin molecules,^68^ and for ‘hardened’ arteries, the use of a K_ATP_ -channel opener (Minoxidil) to increase the elasticity of arteries in a mouse model of Williams syndrome.^69,70^ The latter work has shown evidence of relaxing the arteries, though not yet in relation to cerebral blood flow. For the moment the difficulty in inducing elastin repair^71^ remains a challenge to any quest for healthy aging of the brain.

***The low level of neurogenesis in the adult human brain*.** One hope for an intervention to address age-related cognitive loss lies in the brain's ability to repair itself. What is the prospect that such self-repair might counter dementia?

Two views emerge from the literature. Investigators of neurogenesis stress that there is evidence of both constitutive and regenerational neurogenesis in the adult mammalian brain,^72^ and improved techniques have shown the detail of that neurogenesis in laboratory rodents (especially mice) and in humans.^73,74^ Neurons generated in the dentate gyrus of the hippocampus of adult rat and human have been shown, for example, to achieve the morphology of granule cells. The adult-born neurons have been shown to express a range of neuronal markers; and sites of neurogenesis, initially thought to be restricted to the subventricular zone and the hippocampus of the forebrain, have been identified in the hypothalamus, striatum, amygdala and substantia nigra. With that regenerative potential available, the hope is to upregulate that capacity, to produce sufficient new neurons to offset the age-related damage to brain circuitry that underlies cognitive loss.

A rather less hopeful outlook emerges from a comparative analysis of neurogenesis among vertebrates.^75–77^ Within the class *Mammalia*, for example, adult neurogenesis is more limited than in earlier-evolved classes (best-studied are amphibia and fish); among mammals, adult neurogenesis is more limited in primates than in other orders; and among primates, adult neurogenesis is considered to be least prominent, among the species studied, in *H. sapiens*.^78^ Further (and this is an individual rather than a comparative trend), neurogenesis slows with the age of the individual.^79^ These considerations arguably explain the clinical reality of dementia—that it is still, in practice, so difficult to reverse, stabilize or even slow, once diagnosed.

These two lines of evidence, one promising for self-repair and the other discouraging, have naturally prompted debate. There is an association between an individual's mental activity and dementia,^80^ from which a concept of cognitive resilience or reserve^81^ has developed, re-formulating the hope that our brain can, perhaps with the stimulus of mental exercise, repair its own cognitive performance. The human brain is already remarkable in its ability to function at a high cognitive level for 8 decades and more, with relatively little capacity for adult neurogenesis; self-repair would extend its longevity, and greatly relieve human suffering, For the moment, the clinical reality remains grim: once dementia is diagnosed its progress seems relentless.

***Failure of the blood-brain barrier, including hemorrhage.*** Most pathogens that infect the body do not reach the brain, kept out by the blood-brain barrier (reviewed^82^). A few have, however, evolved ways of penetrating that barrier, by exploiting transport mechanisms of the endothelium of cerebral capillaries to pass through endothelial cells, or to pass between them despite the tight junctions between them, or to infect host phagocytes that pass between endothelial cells.^83^ In addition, and in our view importantly, blood-borne pathogens can ‘ride’ a spill of blood through a breach in a cerebral vessel's wall; so, a hemorrhage.^6^

There is considerable evidence that the infection of cerebral neurons by such pathogens contributes to the cause of dementia. A dementia caused by the syphilis spirochete (a form of bacterium) has been recognized for over a century,^84^ occurring in late stages of infection, typically two decades after an infection that has gone untreated. More recent authors have noted that, in its neuropathology and in the resulting dementia, syphilitic dementia has much in common with Alzheimer's disease.^85,86^ Syphilis is cured by antibiotics, and a timely cure prevents the dementia. The view that this pathogen induces an Alzheimer-like dementia is not disputed.

Balin and colleagues^87^ reviewed extensive evidence that the C*hlamydia pneumoniae* bacterium is causal in human dementia, noting that the bacterium was more prevalent in a series of Alzheimer's disease brains examined postmortem, than in a control group (90% versus 5%). They went on to show that the infected cells included astrocytes, microglia and neurons; that chlamydial infection sites were closely related to Aβ-rich deposits (plaques) and that chlamydial infection was greater in individuals carrying the *APOE4* allele of the *APOE* gene, which predisposes to dementia. In short, the bacterium is highly correlated with several features of the pathology of dementia. It is normally (like other pathogens) excluded from the brain by the blood-brain barrier but, when the barrier is breached by a hemorrhage, *C. pneumoniae* can enter the neuropil and, presumably, can infect brain cells directly.^6^ A second route of entry, demonstrated in mice, is via the olfactory and trigeminal nerves that innervate the nasal cavity.^88^ Once infected, neurons are vulnerable to be killed by the body's immune system, either by killer T-cells that are also normally kept out of the neuropil by the barrier but presumably can enter by ‘riding’ a hemorrhage (argued in^6^), or by components of the brain's innate immune system, including activated microglia.^87^ Although the mechanism of death of infected neurons is not fully established, the association between dementia and the presence of *C. pneumoniae* in the brain seems strong.

Cognitive loss consequent to infection with the human immunodeficiency virus is well recognized.^89^ Treatment with anti-retroviral therapy has been reported to reduce the prevalence of cognitive disorders in the HIV-infected by as much 50%,^90^ thus providing strong evidence that the viral infection is causal. Similarly, an association between dementia and infection by the herpes simplex virus (HSV) has been described.^91,92^ In a Taiwanese cohort, the prevalence of dementia was 2 to 3-fold higher in those carrying an infection. Further, among those with a history of infection, the prevalence of dementia was greatly (∼90%) reduced in individuals who had been treated with anti-viral drugs. Similarly, it has been argued that the *Varicella zoster* (chickenpox, shingles) virus (VZV) may contribute to the cause of dementia by activating the HSV.^93^ A recent study of a Korean cohort of HSV-infected people^94^ reported that HSV and VZV infections increase the risk of dementia and that the risk is raised further in individuals co-infected with the two viruses. In separate studies, evidence is growing that vaccination for shingles (so against the VZV) reduces the individual's risk of subsequently developing dementia.^95,96^

While the data sets that support the idea that pathogens contribute causally to dementia are arguably incomplete, especially as to mechanism, these reports taken together seem compelling.

## Evolved responses to these vulnerabilities: Providential and reactive

One measure of the reality of the threats to the aging human brain just argued is that responses to the threats have also evolved; we listed 12 in.^82^ Some of these protections are providential/preventative. The skull protects against piercing of the brain; the cerebrospinal fluid in the subarachnoid space protects against ‘rocking and rolling’. Sensors in the carotid body and sinus protect against swings in blood pressure and pO_2_. Oxygen is stored in brain cells (as neuroglobin) in case of hypoxia, glucose (as glycogen) in case of hypoglycemia. Autoregulation protects the brain by keeping local blood flow constant. And, in the present context, the elasticity of the walls of the aorta and great distributing vessels in youth is a providential protection against the pulsatility of the heart, because it softens the impact of the pulse on cerebral vessels.^8–10^ Further, the threat of glutamate-induced cytotoxicity is mitigated by the upregulation of glutamate transport out of the brain, into the blood (above). Also noted above is containment of the toxicity of heme by the internalization of hemoglobin within red cells, the restriction of red cells to the bloodstream and the breakdown of red cells by specialized erythrophagocytes in the spleen and liver.

Other protections have evolved to act *post hoc—*after a hemorrhage or other injury. These include the mitigation of the toxicity of hemoglobin by the hypoxia-induced, local expression of first-responders, heme-detoxifying molecules (haptoglobin, hemopexin, the heat-shock protein heme-oxygenase 1 and Aβ)^24^; and, to the extent that it occurs (still debated for humans, above), by adult neurogenesis. The low level of neurogenesis in the human brain suggests that, if cognition is to remain at a high level into old age, neurons must be protected from immune-mediated cytotoxicity. To achieve this, the blood-brain barrier has evolved, able to keep both pathogens and immune cells out of brain tissue, a protection lost when a cerebral vessel bleeds into brain tissue.^6^ In addition, the stress-induced mechanisms of acquired resilience upregulate membrane integrity and mitochondrial function in case of a damaging change, like hemorrhage.^97^

### Another evolved protection: Tissue reserve

The idea that the human brain has a reserve of cognitive ability, and, less clearly argued, of brain tissue, has been proposed and debated for at least two decades, to explain the maintenance of cognitive function in people already affected by what was (and is still by many) considered to be the disease process of Alzheimer's disease, the formation of Aβ^+^ plaques.^81,98,99^ In recent reviews,^25,100^ we have added an argument by analogy, that in those of our organs that cannot self-repair, at least to keep up with age-accumulated damage, function is maintained by an excess of tissue, laid down during individual development. This excess is best established for the kidney. People who donate a kidney for transplantation survive with little effect on longevity,^101,102^ as do people born with only one kidney.^103^ The literature on the effect of losing lung tissue is more limited but similar in conclusion. Bowdish and colleagues^104^ followed 253 people who had donated lobes of a lung for transplantation and reported low morbidity and no mortality among them. A decade later, Chen and colleagues^105^ reported on 85 donors in a Japanese program, also reporting low morbidity and no mortality. Perhaps because transplantation was developed later for the lung, we did not find reports of the effect of lung donation on the longevity of donors. These excesses of functional tissue laid down during development might be termed ‘renal tissue reserve’ (which presumably contributes to ‘renal functional reserve’^106^); and ‘pulmonary tissue reserve’ (which presumably contributes to ‘pulmonary reserve’^107^). Below we develop earlier ideas of brain reserve by asking where is it located? And what is the relation of brain tissue reserve to the onset of dementia?

### Location: Where is brain tissue reserve?

The brain shrinks in every aging individual. Its growth during gestation, childhood and youth reaches a maximum in our twenties (Figure 1) and, beginning in mid-life, that growth reverses and the brain loses weight, the loss arguably accelerating until, in late age, the brain loses 2–3 grams weight per annum.^108^ There seems to be no evidence that any part of the brain is spared from that shrinkage, although there is regional variation in the degree of shrinkage.^109^ Earlier studies suggest that the component of brain shrinkage that is critical for loss of cognition is the thinning of the cerebral cortex, especially of the prefrontal cortex for executive function^110^ and of the temporal lobe and hippocampus for memory. The thinning of the cerebral cortex can be detected by middle age and “spans widespread cortical regions”.^111,112^ In our youth, cortical thickness varies across the surface of the hemisphere from 1.8 to 4.0 mm, with an average of ∼2.8 mm.^111–113^ The rate of thinning over the latter half of human life has been estimated at 0.005–0.01 mm p.a.,^113^ so 0.18–0.36% p.a., averaged over the hemispheres; and up to 4-fold faster in the precentral gyrus (primary motor cortex) and medial prefrontal cortex. By the ninth decade of life the total thinning of the cerebral cortex, still compatible with normal cognition, is in the order of 0.2–0.4 mm.

**Figure 1.**
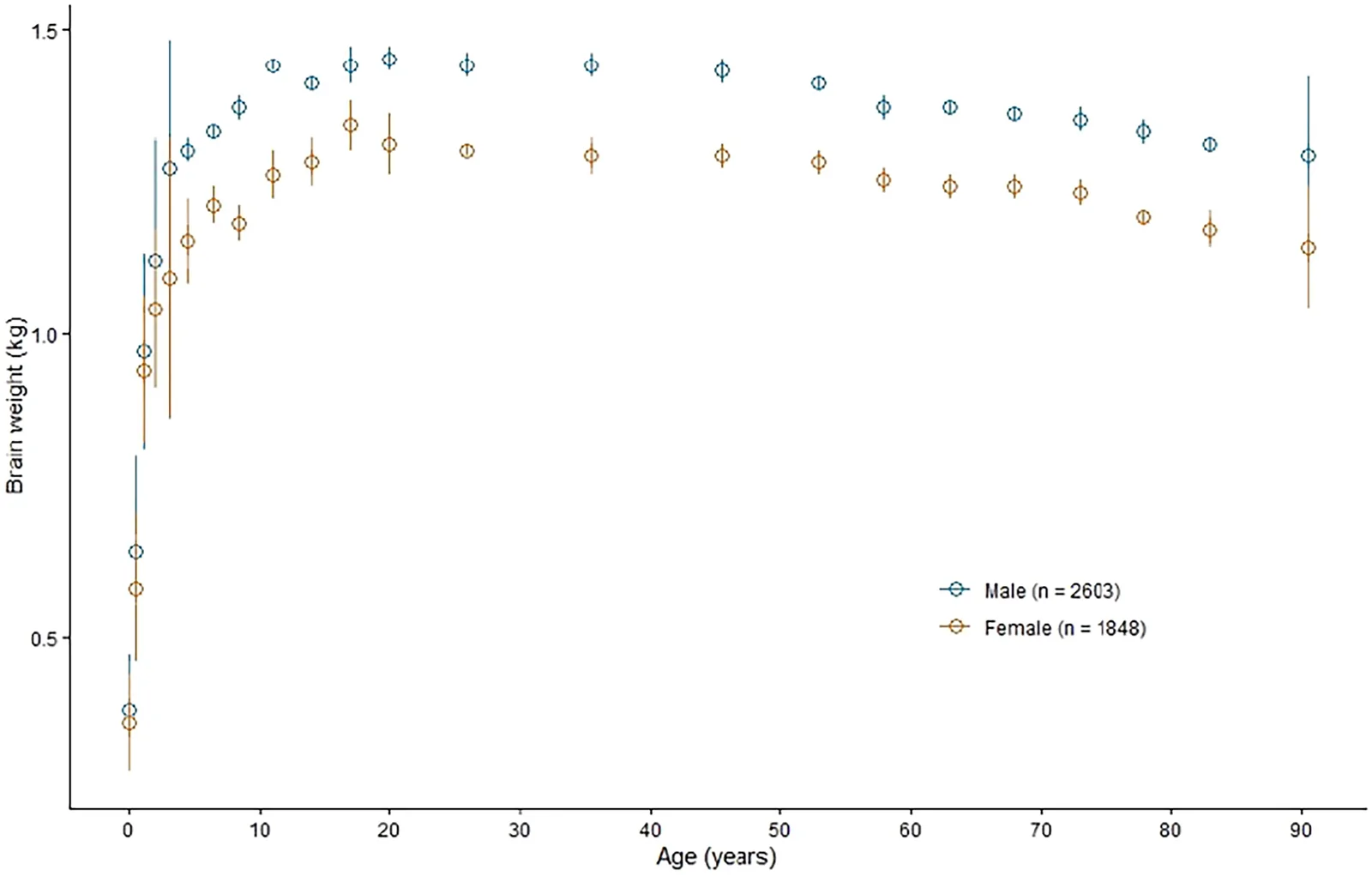
Brain weight as a function of age: data are from Tables 2 and 3 in.^114^ Three features of the graph deserve comment:

*The consistent male-female difference in adult brain weight is at least partly related to differences in body weight. The same difference was reported by.* ^115^

*In both male and female data, brain weight grows to a broad maximum between 20 and 40 years of age and then declines steadily towards the oldest ages. The authors did not comment on whether the cohorts included or excluded people with cognitive loss. The error bars show ± SD. The continuing fall in brain weight to the oldest ages was confirmed in.* ^115–118^

*In the data graphed in Figure 1, variability in weight was narrow in mid-aged adulthood but high during postnatal development and in the oldest age group (86+). The neonatal variability may reflect the rapidity of developmental processes relative to the width of the age range (e.g., 0–10d for the earliest data point). The late variability (at 86 + years) may reflect the variation in cognitive status of people in the 86 + group. This high variability in late life was confirmed in* ^118^ *but not in,* ^116,117^ *and deserves further investigation.*

Further, Pacheco and colleagues^113^ and Roe and colleagues^119^ reported evidence that unusually rapid thinning of the cortex is a predictor of subsequent dementia. And conversely, several groups have reported that exercise slows the rate of cortical thinning (including in cognition-critical prefrontal cortex), so that older exercisers have thicker cerebral cortex than age-matched sedentary people; and, correspondingly, exercise delays the loss of cognition.^120–122^ The thinning and the rate of thinning of the cerebral cortex are then, we suggest, measures of depletion of the brain tissue reserve. We take the concept one small step further: when the reserve becomes exhausted, we suggest, the tragedy of dementia begins.

The evolutionary pressures that led to the laying down of reserve tissue in kidney, lung and brain, despite the metabolic cost of generating the reserves and maintaining them for decades, have not (as far as we could find) been discussed in the literature. Why did these reserves of tissue not evolved to be sufficient for cognition to last as long as the rest of the body, so that the gap between the life of the brain and body, the period of dementia, was minimal? And so reduce the years of cognitive decline (∼2 years in men diagnosed in their 80 s, as many as 9 years in women diagnosed in their 60s^123^)? Perhaps this gap has occurred because the late decades of human life are long after child-rearing, so there was less evolutionary pressure for the development of further tissue reserve.

## Dementia and the mechanisms of antagonistic pleiotropy (AP)

AP is a phenomenon of the evolution of species first identified (described^124^ and named^125^) in the middle of the last Century. It has been defined in several ways, perhaps most simply as the evolution of a phenotype which is beneficial for a species during youth and fecundity, but in age is seemingly damaging (antagonistic) to the individual animal and perhaps to the species. AP is, arguably, one of the ‘best-accepted’ mechanisms of aging^126–128^ and has been investigated by modern techniques like genome-wide analysis, which have sought to identify the genetic actions involved^129^ in this complex phenotype.

The present analysis of the evolutionary origins of dementia is arguably a ‘text-book’ case of AP, in which evolutionary changes empower the young individuals, but cause the tragedy of dementia in the aging. The analysis is made, however, in terms of changes in tissues—capillary hemorrhage, the toxicity of heme, the failure of elastin to regenerate in the walls of the aorta and more. Many tissues are involved and presumably many genes.

Others have queried the meaning of AP-driven aging. Is AP a ‘mechanism of aging’? Does it benefit the species by clearing out the old to create space for the next generation? We note that, in humans, dementia occurs decades after fecundity, and that the suffering inflicted on individuals and their carers is extreme. Dementia may ‘deal with’ the old who live too long for the good of the young, but it does so late, and cruelly.

### Evolution and the inevitability of dementia

The present considerations bear on a lingering question about the tragedy of dementia: Is it a normal part of human life, one of the ‘natural causes’ of death? Or is it a disease? The alternatives have different implications for research, drug development and the individual (see Figure 2 in^100^). If dementia is a **disease** with perhaps a cure, then the individual waits to see if they suffer it and then seeks a post-diagnosis cure. Research, grant funding and marketing then focus on drugs that have the promise or at least the rationale to provide a cure. If dementia is a **fate**, the individual seeks ways to delay it. Researchers seek to identify pre-diagnosis/lifestyle measures (diet, exercise^97^ and more^80^) that delay, and things to avoid (head and brain trauma) and research and grant funding are directed to these very different questions. The difference has great implications for all parts of research and treatment. So, which is it? A curable disease or a fate?

Historically, awareness of dementia is clear in writings from times as distant as two millennia, including Pythagoras (reviewed^130–132^), Lucretius^133^ and Hua Tuo.^134^ The oldest description may be a brief reference in the Ebers Papyrus (c. 1550 BC, so the best part of a millennium before Pythagoras) to ‘perishing of the mind and forgetfulness’.^135^ Pythagoras and Lucretius seem (lines 459–468 in^133^) to have considered dementia to be the last stage of a normal life; which Pythagoras (paraphrased in^131,132^) estimated to start at the age of 81. A millennium and more later, Shakespeare described the last of the ‘seven ages of man’ as *second childishness and mere oblivion*, more recently termed senile dementia. This view continued until Alzheimer's reports, early in the twentieth Century.^43,136^ Arguably the most important element of these reports was not Alzheimer's descriptions of cognitive breakdown and its neuropathology, both had been described previously, but his observation of dementia occurring in mid-life. Such early-onset dementias must, he argued, be ‘eigenartige’ (distinctive), and not normal life. Kraepelin included Alzheimer's observations in the 10^th^ edition of his *Lehrbuch der Neurologie*^137^ under the heading *Alzheimer's Krankheit* (disease). And ‘disease’, unlike ‘senility’, implies the possibility of cure.

The search for a cure was accelerated in the later decades of the twentieth Century by the power of molecular biology. A 2006 volume of over 50 papers,^138^ and other volumes from the same period (e.g.,^139^) recounted and justifiably celebrated remarkable advances in the understanding of the aging brain achieved in those decades. In the present context, we make three comments. First, those involved in these discoveries understood that they were attempting ‘the biomedical deconstruction of senility’^140^; they sought the mechanisms that cause dementia, and then ways to cure it, to reverse it or at least halt the loss of cognition. Second, in the two decades since that volume, the hope of understanding and treatment has faded. The genetic basis of early (mid-life) onset dementia was established, shown to lie in high-penetrance mutations in three genes related to amyloid precursor protein; but little if any advance has been made towards treatment.^141,142^ And for the more common, late-life/sporadic form of dementia, which has come to be included in the term Alzheimer's disease, there seems to be neither agreement on mechanism nor clinically significant progress in treatment.

Senile dementia seems for the moment to have defied deconstruction, to be an exception to the optimistic view that every challenging concept is solvable (Chapter 3 in^4^). With the hope of understanding and cure frustrated, the effort to deconstruct senility has produced controversies concerning commercial claims and regulatory rulings. The recent regulatory approval of one drug for the treatment of dementia (Aduhelm, Chapter 6 in^4^), for example, has been described as ‘more a symbol of desperation ……. than real progress’ and it is possible that frustration and disappointment created conditions for the damaging laboratory fraud which is the main theme of Piller's 2025 book.^4^ To some investigators, but far from all,^143^ it seems more appropriate at least for the time being still to view late-life dementia as normal aging, normal because the causes of the associated degeneration of the brain are so entrenched in our evolution and physiology*.*

Our third comment concerns organs other than the brain. We have argued previously^25^ that the failures in old age of three organs—lung, kidney, and brain—are all caused by pulse-induced capillary hemorrhage, exacerbated by the hypertension of age. Part of the evidence linking their failures (which produce idiopathic pulmonary fibrosis, chronic kidney disease and dementia) is that all three organs are highly vascular, all show evidence of microbleeds in late life, none can significantly self-repair, all undergo slow shrinkage from mid-life yet maintain function within normal levels by deployment of a tissue reserve, the risk of all three is increased by hypertension and they commonly fail together. If their cause lies in the unmodifiable evolutionary and vascular factors discussed above and in,^6,10,14,100^ then none of these three conditions is a curable disease; rather they are the way we die, if accidents or warfare or infectious or non-infectious disease do not claim us first. This returns us to the view of earlier millennia, that dementia is a normal part of life. This is a bleak view, but likely one that must eventually be accepted. And that acceptance may^144^ reduce the frustration and disappointment that triggered the fraud that in turn distorted some brilliant science, and will allow us to focus on what is possible—the delay of dementia by the preservation of brain tissue reserve.^14,81,100^

## To what age can brain tissue reserve last? Data on the incidence and prevalence of cognitive loss

If dementia is a fate, then the prevalence of dementia should increase with age towards 100%. Confirming this prediction, Brookmeyer and colleagues reported that the incidence of new cases of dementia increases exponentially with age, with a doubling time of ∼5 years from a low base (<1%) in the years 60–65.^145^ They assessed their cases (combined from four studies of the US population) in 5-year steps through to 94–99 and noted that the pattern predicts ‘very high incident rates’ at still older ages. Subsequent studies of the ‘oldest-old’^146,147^ confirmed this prediction, reporting incidence rates of 40.7% and 48.2% at 94–99 and in centenarians respectively, and prevalences of 39.6% at 94–99 and 65% in centenarians. Consistent with this, Leung and colleagues^148^ reported that, in a cohort of people aged 95 or more, ‘*dementia prevalence was 53.2% in women and 45.5% in men, with risk continuing to increase with age’.* Unless there is some slowing of dementia incidence in the 12^th^ decade of life, prevalence at that age will approach 100%.

## Conclusion: The evolutionary causes of dementia

Despite all the protections that have evolved to counter its vulnerabilities, the human brain becomes dysfunctional in advanced age, and the prevalence of this dysfunction (dementia) increases exponentially with age. Medical science and the genius of health professionals have extended human longevity, with the result that age-related neurodegenerations have reached ‘plague proportions’^26^ in the very old. This essay has focused on evolutionary causes of dementia, of which we identify seven, as set out in Table 1.

**Table 1.** Seven evolutionary features of human evolution that contribute to neuronal death, and therefore to dementia, in the aging human brain.

Evolved feature	Advantage in youth	Disadvantage for brain
**Three features related to oxygen and its delivery**		
Use of oxygen (O_2_) to maximize production of cellular energy (ATP) from food. ^27,28^	O_2_-fuelled metabolic pathways provided a huge boost to the energy of animals, such as vertebrates.	The brain is so dependent on O_2_ that it is always only seconds of anoxia away from failing and a few minutes away from irreversible damage.
Heme, as oxygen carrier^6,24,31–35,37,38,40^	Most efficient evolved O_2_ transporter. A transporter is essential for the O_2_-demanding metabolism of vertebrates.	Heme is toxic to all tissues; must be enclosed in specialized (red) cells, in blood. Hemorrhage unleashes heme's neuron-destructive toxicity.
Pulsatility of the heart^21,22,41,42,44–51,136^	A contractile/beating heart is the only mechanism that has evolved to circulate blood in animals. For many humans, the heart functions robustly for many decades. In a lifespan of 80 years, it beats ∼3 billion times.	Each contraction of the heart produces a pulse whose intensity increases with age, as artery walls harden. The sharpening pulse bursts cerebral vessels, causing hemorrhages. Neurons in affected areas are damaged/ killed. The cumulative result is dementia.
**Excitotoxicity**		
Glutamate as the brain's major excitatory neurotransmitter.^52–57^	Glutamate is the brain's most abundant excitatory neurotransmitter. Its evolution involved the repurposing an amino acid readily available in food.	Glutamate transporters in the walls of blood vessels reduce glutamate in the brain to the levels needed for signaling. If/when they fail, glutamate levels in the brain rise and kill neurons by over-excitation.
**Three causes from failure of repair or of protection**		
Elastin in the aorta^58–63,65–69,132,149^	A thick elastin layer in the walls of great arteries buffers each systole and maintains flow through diastole. This ‘smooths’ the pulse, making it less damaging.	Elastin failure with age contributes to hypertension, a major risk for dementia, increasing the likelihood of pulse-induced hemorrhages in cerebral vessels.
Low neurogenesis in adult brain of humans.^72–81,89^	For reasons not understood, neurogenesis in the adult human brain is much less than in earlier-evolved vertebrates (fish, amphibians).	This reduces the ability of the adult human brain to repair itself.
The failure of the blood brain barrier (ΒΒΒ). ^82–89,91–96^	The BBB protects the brain 1) by containing red cells, so preventing heme from contacting neurons and damaging them; 2) by containing opportunistic pathogens (viruses, bacteria), preventing the infection of neurons: and 3) by keeping killer T-cells out of the brain.	With age the BBB breaks down at the site of a hemorrhage, allowing 1) heme into the neuropil where it causes damage to neurons; 2) pathogens to reach and infect neurons and 3) killer T-cells to reach and kill infected neurons.

The credibility of the idea of evolutionary origins of dementia argued above is strengthened by the protections evolved to mitigate each. The recognition of evolutionary causes and protections does not seem likely to change the treatment of dementia once diagnosed, still less to produce a restoration of cognition, something, we argue, that is not attainable after the exhaustion of brain tissue reserve. But evolutionary understanding may help the acceptance that the best treatment for dementia is^100^ to delay its onset by providential choices, implemented well before diagnosis, to ‘preserve the reserve’.
